# Design of Polymeric Delivery Systems for *Lycium barbarum* Phytochemicals: A Spray Drying Approach for Nutraceuticals

**DOI:** 10.3390/foods14203504

**Published:** 2025-10-15

**Authors:** Filipa Teixeira, Angelina Rut, Paulo C. Costa, Francisca Rodrigues, Berta Nogueiro Estevinho

**Affiliations:** 1 REQUIMTE/LAQV—Rede de Química e Tecnologia/Laboratório Associado para a Química Verde, ISEP—Instituto Superior de Engenharia do Porto, Politécnico do Porto, Rua Dr. António Bernardino de Almeida, 4249-015 Porto, Portugal; 2 ICBAS—Instituto de Ciências Biomédicas Abel Salazar—School of Medicine and Biomedical Sciences, University of Porto, Rua de Jorge de Viterbo Ferreira, 228, 4050-313 Porto, Portugal; 3 Gheorghe Asachi Technical University of Iasi, 67 prof.dr.doc. D.Mangeron Street, 700050 Iasi, Romania; 4 UCIBIO—Applied Molecular Biosciences Unit, MedTech-Laboratory of Pharmaceutical Technology, Faculty of Pharmacy, University of Porto, Rua Jorge Viterbo Ferreira, 228, 4050-313 Porto, Portugal; pccosta@ff.up.pt; 5 Associate Laboratory i4HB—Institute for Health and Bioeconomy, Faculty of Pharmacy, University of Porto, Rua Jorge Viterbo Ferreira, 228, 4050-313 Porto, Portugal; 6 LEPABE—Laboratory for Process Engineering, Environment, Biotechnology and Energy, Faculty of Engineering, University of Porto, Rua Dr. Roberto Frias, 4200-465 Porto, Portugal; 7 ALiCE—Associate Laboratory in Chemical Engineering, Faculty of Engineering, University of Porto, Rua Dr. Roberto Frias, 4200-465 Porto, Portugal

**Keywords:** controlled release systems, goji berries, functional foods, polymeric microparticles, spray atomization technique

## Abstract

Goji berries (*Lycium barbarum* L.) are extremely rich in bioactive compounds, including phenolics, flavonoids, and vitamin C, which contribute to the strong antioxidant and immunomodulatory properties, positioning them as a promising candidate for nutraceutical applications. However, due to some limitations such as poor bioavailability and instability, encapsulation via spray drying with polymeric carriers provides a practical strategy to improve their stability, bioavailability, and applicability in the health sector. In this study, goji berry extract (GBE) was obtained via ultrasound-assisted extraction (UAE) and encapsulated using spray drying with four different polymers: alginate, pectin, Eudragit E100 and RS30D. GBE-loaded microparticles showed improved production yields (e.g., 40.3% for Alginate + GBE vs. 13.9% for Alginate alone) and varying particle sizes (1.9–4.4 µm). The antioxidant/antiradical activities were retained to different extents, depending on the carrier, with RS30D + GBE displaying the highest TPC (15.51 mg GAE (gallic acid equivalents)/g), FRAP (59.83 µmol FSE (ferrous sulphate equivalents)/g), and DPPH activities (3.50 mg TE (Trolox equivalents)/g). Biocompatibility was confirmed in HT29-MTX cell lines for all produced microparticles. These findings support the use of spray-dried polymeric carriers to enhance the functional performance and stability of goji berry bioactive compounds in future nutraceutical applications.

## 1. Introduction

*Lycium barbarum* L., commonly known as goji berries, is a fruit deeply rooted in traditional Asian medicine and cuisine, praised for its remarkable health-promoting properties [1,2]. Originally native to China, goji berries are now widely cultivated across other parts of Asia and are increasingly grown in Europe and North America, gaining global recognition as a “superfruit” [1]. Their growing popularity stems from a rich phytochemical profile, especially high concentrations of phenolic compounds (12697.90 mg/100 g fw), organic acids (4461.02 mg/100 g fw), vitamin C (2.39–48.94 mg/100 g fw), flavonoids (37.60 mg/g dw) and carbohydrates (77.1–87.0 g/100 g dw) [1,2,3]. These bioactive constituents are primarily responsible for the antioxidant, anti-inflammatory, antimicrobial, neuroprotective, and immunomodulatory activities attributed to goji berries [1,4]. Scientific research has confirmed that goji berries extracts possess potent antioxidant capabilities, primarily through their ability to neutralize reactive oxygen and nitrogen species (ROS and RNS, respectively) [5], regulate oxidative stress markers, and enhance endogenous antioxidant defense systems [6,7]. These properties suggest that goji berries could be highly effective in preventing or mitigating chronic diseases linked to oxidative damage, such as cardiovascular disease, cancer, neurodegenerative disorders, and aging-related conditions [6]. Furthermore, immunomodulatory studies have shown that certain extracts can enhance immune responses, promoting natural killer cell activity and modulating cytokine levels [7].

Despite the promising therapeutic potential of goji berries phytochemicals, their direct application in food, pharmaceutical, or nutraceutical products is still limited. Challenges related to poor bioavailability, chemical instability, degradation during digestion, and limited intestinal permeability are well reported [8,9]. To overcome these drawbacks, polymeric delivery systems have emerged as a valuable strategy [7,9]. These systems consist in the encapsulation of bioactive compounds within biodegradable and biocompatible polymers that protect them from environmental stressors and control their release, while improve their solubility and absorption [10,11]. Among the various encapsulation techniques, spray drying is particularly attractive due to its scalability, cost-effectiveness, and suitability for heat-sensitive ingredients, as it converts a liquid extract into a dry powder by atomizing it into a hot air stream [9,12]. When combined with appropriate carrier materials, the active compounds can be embedded into microparticles that serve as protective matrices. This process offers several advantages: (i) it preserves the functional integrity of sensitive compounds, (ii) masks unpleasant flavors, and (iii) produces a stable, easily handled powder form that can be readily incorporated into capsules, tablets, sachets, or functional foods [7,12]. In the nutraceutical field, the development of such delivery systems is highly relevant, as the demand for natural, plant-based functional ingredients continues to grow, driven by health-conscious consumers seeking alternatives to synthetic drugs [8,12]. Goji berries, with their well-documented bioactivities, are ideal candidates for nutraceutical formulations.

Therefore, the aim of this work is to integrate advanced extraction techniques, namely ultrasound-assisted extraction (UAE), with spray-dried polymeric encapsulation to develop goji berry-based nutraceuticals that are both scientifically validated and commercially viable. In the present study, four polymers with distinct chemistries and release profiles were selected, namely sodium alginate and pectin (natural, anionic, water-soluble polysaccharides) [8,13], Eudragit E100 (a cationic, pH-dependent polymer) [14], and Eudragit RS30D (a water-insoluble, pH-independent polymer) [15], to systematically investigate how carrier type affects encapsulation efficiency, particle characteristics, and biocompatibility. This approach bridges the gap between promising laboratory results and consumer-ready nutraceutical products, unlocking the full potential of goji berries in human health and nutrition.

## 2. Materials and Methods

### 2.1. Chemicals and Reagents

Eudragit RS30D and Eudragit E100 were kindly supplied by Evonik Operations GmbH (Darmstadt, Germany). Sodium alginate (alginic acid, sodium salt) and Thiazolyl Blue Tetrazolium Bromide (MTT) were obtained from Sigma Aldrich (St. Louis, MO, USA). Pectin (Poly-D-galacturonic acid methyl ester)-Apple pectin was obtained from Sigma Aldrich (Buchs, Switzerland). Dulbecco’s Modified Eagle Medium (DMEM), Hanks Balanced Salt Solution (HBSS) and Essential Amino Acids were obtained from Biowest (Nuaillé, France). Penicillin/streptomycin solution and fetal bovine serum (FBS) were obtained from PAN^TM^ BioTech (Darmstadt, Germany). Dimethyl Sulfoxide (DMSO) was obtained from Thermo Fisher (Merelbeke, Belgium). Triton^®^ X-100 was obtained from VWR (Fontenay-sous-Bois, France).

### 2.2. Preparation of Lycium Barbarum Extract

Dried *L. barbarum* berries were sourced from Naturefoods (Porto, Portugal) in October 2022. The berries were milled (Moulinex A320, Moulinex, Écully, France) into a fine powder and stored in a dark place until extraction. The UAE was carried out using an ultrasonic probe processor (Sonic Vibracell, model VCX50, Newtown, CT, USA) fitted with a 13 mm diameter probe tip (No. 630-0219) operating at 20 KHz. The extraction was performed with water as solvent, using a sample-to-solvent ratio of 8.75%, (*w/v*), a probe amplitude of 59%, and 750 W of power, for 56.215 min at room temperature, according to Teixeira et al. [5]. The resulting mixture was centrifuged (Megafuge™ 16, Thermo Scientific, Waltham, MA, USA) at 5000 rpm for 45 min at 20 °C for debris removal, followed by filtertion through Whatman n °1 paper. The clarified extracts were frozen at −80 °C, lyophilized (Cryodos–80, Telstar, Barcelona, Spain) and kept at 4 °C until further analysis.

### 2.3. Preparation of Polymer Microparticles

Spray-drying was employed to produce eight types of microparticles: empty and GBE-loaded Eudragit RS30D (RS30D and RS30D + GBE), Alginate (Alginate and Alginate + GBE), Pectin (Pectin and Pectin + GBE), and Eudragit E100 (E100 and E100 + GBE) formulations, each prepared at 1% (*w/v*). The process was carried out using a BÜCHI Mini Spray Dryer B-290 (Flawil, Switzerland) equipped with a 0.5 mm nozzle, according to Chaumun et al. [16]. All solutions, except the ones produced with Eudragit E100 (1%, *w/v*), were fed into the spray dryer at a flow rate of 4 mL/min (15%) and at an inlet temperature of 115 °C. Air pressure and aspiration rate were set to 5–6 bar and 100% (36 m^3^/h), respectively. The outlet temperature was around 55–60 °C. For the case of samples containing Eudragit E100, a mini spray-dryer B-290 coupled to an Inert Loop B-295, all from BÜCHI (Flawil, Switzerland) was used with nitrogen pressure, aspiration rate, solution feed flow, inlet air temperature and the temperature of Inert Loop B-295 set at 2 bar, 36 m^3^∙h^−1^ (100%), 4 mL∙min^−1^, 90 °C and −5 °C, respectively. The outlet air temperature was around 56 °C. All feed solutions containing extracts were prepared by mixing 5 mL of GBE with 100 mL of the respective polymer solution, based on optimized preliminary tests. The microparticles were prepared with around 30% w/w of extract, except the microparticles prepared with Eudragit RS 30 D, that had around 60% w/w of active compound.

### 2.4. Characterization of the Optimal Liposome Formulation

#### 2.4.1. Production Yield

The production yield was determined as the ratio between the total mass of powder collected from the inner cylinder of the spray dryer and the total mass of extract and/or excipients present in the feed solution, as expressed in Equation (1).

(1) $$Yield\left(\%\right)=\frac{weigth\;of\;the\;collected\;powder}{inicial\;weigth\;ofextract/excipients\;in\;the\;feed\;solution}\times 100$$

#### 2.4.2. Particle Shape, Size, and Surface Properties

Scanning electron microscopy (SEM) was employed to investigate the polymeric microparticles morphology, using a Fei Quanta 400 FEG ESEM/EDAX Pegasus X4M (Eindhoven, The Netherlands). The analyses were carried out in a JEOL JFC-100 apparatus with accelerating voltage of 15 kV and a magnification of 1000×, 10,000×, and 30,000×. Samples were placed in the analyzer by fixating the microparticles in a brass stub with double-sided adhesive tape and then coated in vacuum by a thin layer of gold. The samples were coated to ensure adequate conductivity for high-resolution imaging. SEM images were acquired at multiple magnifications to capture both overall morphology and finer structural details. Several regions of each sample were examined to verify uniform particle distribution and avoid bias. Particle size and morphology were quantified using ImageJ software (v1.54d), with six distinct structures measured per sample to ensure reliable data.

#### 2.4.3. In Vitro Antioxidant and Antiradical Activities

##### Total Phenolic Content

The total phenolic content (TPC) was determined using the Folin–Ciocalteu method, following the procedure described by Singleton and Rossi [17] with minor modifications. Gallic acid was used to construct the standard curve (linearity range: 5–100 µg/mL; *R*^2^ > 0.998), and results were expressed as milligrams of gallic acid equivalents per gram of microparticles (mg GAE/g).

##### DPPH Radical Scavenging Activity Assay

The DPPH free radical scavenging activity was assessed according to the method described by Barros et al. [18], with minor modifications. Trolox was employed as the standard (linearity range: 5–125 µg/mL; *R*^2^ > 0.998), and results were expressed as milligrams of Trolox equivalents per gram of microparticles (mg TE/g).

##### Ferric Reducing Antioxidant Power (FRAP) Assay

The FRAP assay was conducted following the method of Benzie & Strain [19], with minor modifications. Ferrous sulfate heptahydrate (FeSO_4_·7H_2_O) was used as the standard (linearity range: 25–500 µM; *R*^2^ > 0.996), and results were expressed as micromoles of ferrous sulfate equivalents per gram of microparticles (µmol FSE/g).

#### 2.4.4. Differential Scanning Calorimeter (DSC)

Differential Scanning Calorimetry (DSC) analyses were carried out on both the lyophilized extract and the formulated microparticles using a DSC 200 F3 Maia instrument (Netzsch–Gerätebau GmbH, Bayern, Germany). Samples were placed in aluminum pans with perforated lids, and measurements were performed from 0 °C to 200 °C at a heating rate of 10 °C/min. Onset temperatures were determined using Proteus Analysis software (Version 6.1, Netzsch–Gerätebau GmbH, Bayern, Germany).

#### 2.4.5. In Vitro Biocompatibility Studies

HT29-MTX (intestinal epithelial cell) cell line was acquired from American Type Culture Collection (ATCC, Manassas, VA, USA). HT29-MTX (passage 43) were grown in Dulbecco’s Modified Eagle Medium (DMEM), previously supplemented with 10% fetal bovine serum, 1% essential amino acids and 1% antibiotics (penicillin/streptomycin solution). The cells were maintained at standard conditions of a temperature of 37 °C with 5% CO_2_ and the culture medium was changed every two days until reaching confluence.

The biocompatibility of the produced microparticles was assessed by cell viability using the 3-(4,5-dimethylthiazol-2-yl)-2,5-diphenyltetrazolium bromide (MTT) colorimetric assay [20]. In each well of a 96-well plate, the cells were seeded at a concentration of 1 × 10^4^ cells/well and incubated for 24 h at standard conditions in order to provide exponential growth. After the incubation period, the medium was removed, the wells were washed with HBSS (200 µL) and incubated with different concentrations of loaded and unloaded microparticles (31.25–500 μg/mL), dissolved in the respective medium, for 24 h. Afterwards, the MTT (120 µL) was added to each well and incubated for 3 h at standard conditions. Afterwards, the MTT (120 µL) was added to each well and incubated for 3 h at standard conditions. After incubation, the MTT solution was removed, and the resulting formazan crystals were dissolved in 200 µL of DMSO. The absorbance was measured at 570 nm, with background correction at 630 nm, using a Synergy™ HT Multi-mode microplate reader (BioTek Instruments Inc., Winooski, VT, USA). Culture medium served as positive control, while 1% (*v/v*) Triton X-100 was used as the negative control. Cell viability (*CV*) was calculated relative to the untreated control group, which was considered 100%, as described in Equation (2).

(2) $$CV\left(\%\right)=\frac{A_{570}-A_{630}\;treat\;cell}{A_{570}-A_{630}\;untreat\;cell}\times 100$$

### 2.5. Statistical Analysis

All experiments were conducted in triplicate (*n* = 3), and data are presented as mean ± standard deviation (SD). Statistical significance was set at *p* < 0.05, determined by one-way analysis of variance (ANOVA) followed by Tukey’s HSD post hoc test, using IBM SPSS Statistics 27.0 (SPSS Inc., Chicago, IL, USA).

## 3. Results

### 3.1. Characterization of the Produced Microparticles

#### 3.1.1. Production Yield

The production yield varied significantly among formulations and was generally higher when GBE was included, indicating a positive impact of the extract on spray drying performance (Table 1). Spray-drying efficiency is strongly influenced by key feed properties, such as viscosity, surface tension, and solids concentration, which affect droplet formation, drying kinetics, and wall deposition [20]. The presence of GBE likely modified these properties, slightly increasing viscosity and reducing surface tension, resulting in more uniform droplets, improved atomization, and reduced adhesion of particles to the dryer walls, all of which contribute to higher product yield [20].Eudragit E100 alone exhibited an extremely low production yield (2.8%), likely due to its poor spray-drying behavior when used without additional bioactive compounds. However, upon adding GBE, the yield increased substantially to 26.6%, suggesting that the extract act as a solidifying or bulking agent and improving feed solution properties such as viscosity or drying behavior [21]. Alginate-based systems showed moderate production yields, which increased from 13.9% (alginate alone) to 40.3% with GBE. Alginate’s natural gelation capacity and good solubility likely facilitated efficient atomization and drying [8,13]. The addition of GBE appears to improve the matrix formation, possibly due to interactions between alginate and phenolic compounds, enhancing the solid content and the thermal stability during drying [13]. Pectin formulations demonstrated a similar trend, with a relatively high yield of 21.7% without GBE that increase to 38.1% when GBE was present. Pectin’s intrinsic drying properties, combined with its compatibility with polyphenols, likely contributed to improving the drying efficiency [22]. RS30D alone had the lowest yield (1.3%), which is consistent with the poor atomization and film-forming characteristics of this water-insoluble polymer [15]. However, when combined with GBE, the yield improved notably to 20.1%. This reinforces the hypothesis that the presence of GBE alters the drying kinetics or feed properties, improving the retention of solid material.

Overall, the spray drying of GBE with natural (alginate, pectin) and synthetic (Eudragit E100, RS30D) polymers attested that GBE significantly enhanced the production yields across all polymer systems, likely by improving the solid content, feeding viscosity, or promoting the particle formation during drying [23].

#### 3.1.2. Microstructure Size

Physical characteristics of spray-dried microparticles are key parameters in developing functional delivery systems. As shown in Table 1, all the microstructures exhibited a mean diameter ranging from 1.9 to 4.4 µm. Eudragit E100 produced small particles (3.3 µm) and the size remained consistent (2.9 µm) with GBE, indicating that this polymer forms fine particles regardless the presence of actives. Alginate and Pectin systems showed slightly larger particles (3.2–4.1 µm). Notably, Pectin + GBE produced the largest average particles (4.1 µm), possibly due to pectin’s high molecular weight and viscosity that can affect the droplet size during atomization [24]. The increase in particle size upon GBE addition may also be attributed to matrix swelling or the presence of higher solute content. RS30D alone produced the largest particles (4.4 µm), despite the particle size decreased dramatically to 1.9 µm when GBE was incorporated. This unusual result may be related to the GBE’s influence on feed dispersion or a plasticizing effect that alters the solidification rate, resulting in smaller, more compact particles [15].

#### 3.1.3. In Vitro Antioxidant and Antiradical Activities

Table 2 summarizes the antioxidant and antiradical activities of the GBE and the produced microparticles evaluated by TPC, FRAP and DPPH assays. GBE alone exhibited the highest antioxidant/antiradical potential across all assays, being consistent with its richness in phenolic compounds and bioactive metabolites such as flavonoids, carotenoids, and polysaccharides [5]. The encapsulation of GBE with different polymers led to an overall reduction in the antioxidant/antiradical activities, although the degree of reduction varied considerably depending on the polymer used. Eudragit E100 alone showed the lowest values across all assays (TPC: 3.53 mg GAE/g; FRAP: 5.10 µmol FSE/g; DPPH: 0.94 mg TE/g), indicating minimal intrinsic antioxidant/antiradical capacities. When combined with GBE, the antioxidant/antiradical values increased but remained substantially lower than pure GBE. This suggests the probably chemical interaction or limited protection of polyphenols during spray drying with E100, possibly due to its cationic nature and low permeability, which might restrict the release or accessibility of GBE compounds [14]. On the other hand, encapsulation with alginate (a natural, anionic polysaccharide) led to better retention of the antioxidant/antiradical properties. The Alginate + GBE formulation showed moderate antioxidant/antiradical activities (TPC: 7.85 mg GAE/g; FRAP: 42.16 µmol FSE/g; DPPH: 3.33 mg TE/g), suggesting the effective entrapment and partial protection of bioactive compounds. Alginate has a well-known ability to form hydrogels and its mild encapsulation conditions may help to preserve phenolic compounds during spray drying [8,13]. Pectin, a plant-derived polysaccharide, also preserved the antioxidant/antiradical activities reasonably well (TPC: 8.14 mg GAE/g; FRAP: 43.20 µmol FSE/g; DPPH: 4.14 mg TE/g). Its performance was similar to alginate, likely due to its gelling ability and compatibility with polyphenols. Pectin may form a protective matrix that reduces the oxidation and thermal degradation during spray drying, especially for heat-sensitive goji berry compounds [24]. Regarding the RS30D polymer, a water-insoluble but permeable copolymer, it exhibited the best performance among synthetic matrices. The RS30D + GBE formulation retained higher TPC (15.51 mg GAE/g), FRAP (59.83 µmol FSE/g) and DPPH (3.50 mg TE/g) values compared to other encapsulated forms. This suggests that RS30D provides a more effective barrier against the oxidative degradation, while allowed a controlled release of the bioactive compounds [15]. Its partial permeability could facilitate the diffusion of bioactive compounds during the assays, while still providing significant protection in the process.

#### 3.1.4. Surface Morphology

**Figure 1:**
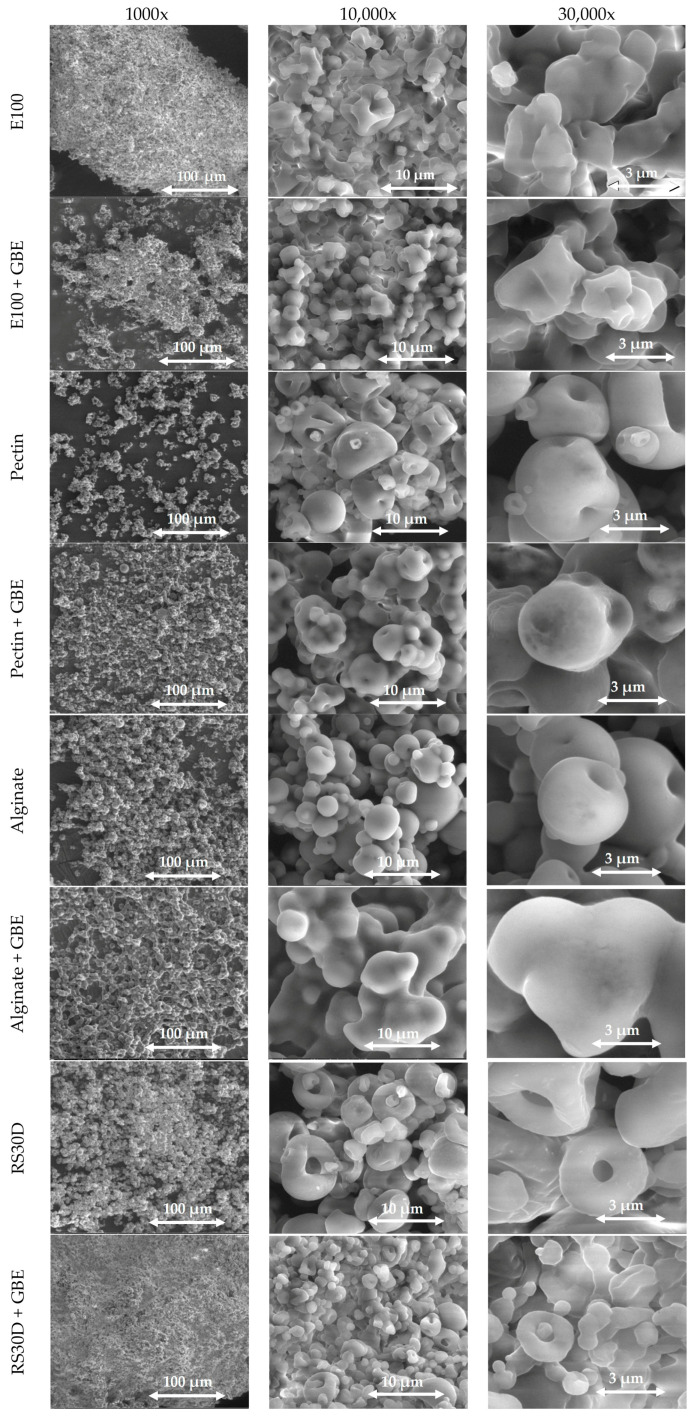
SEM images of spray-dried microparticles at three different magnifications: 1000×, 10,000×, and 30,000×. Each row corresponds to a specific formulation, either using a single polymer (Eudragit E100, Alginate, Pectin, or Eudragit RS30D) or the same polymer combined with GBE. Beam intensity (HV) of 10.00 kV.

SEM analyses are represented in Figure 1. All the produced microparticles display a spherical morphology, with a heterogeneous size distribution and with a different degree of rugosity of the surface. Microstructures with different morphologies were obtained depending on the encapsulating agent used. Microstructures with a curious “donut” appearance were formed with Eudragit RS30D with and without extract [20]. In general, the addition of the extract to microstructures increases the adherence between particles and reduces the spherical definition of the microstructures. This fact is more relevant for the microparticles prepared with alginate. However, the particles prepared with pectin also registered a considerable increase in the adherence with the addition of the extract. Therefore, the addition of the Goji extract provoked the formation of more amorphous structures with a high agglutination between particles, namely in the microstructures prepared with alginate and pectin. The structures prepared with Eudragit appeared to be more resistant to the effect of the extract addition in the microparticles morphology.

#### 3.1.5. Thermal Behavior

**Figure 2:**
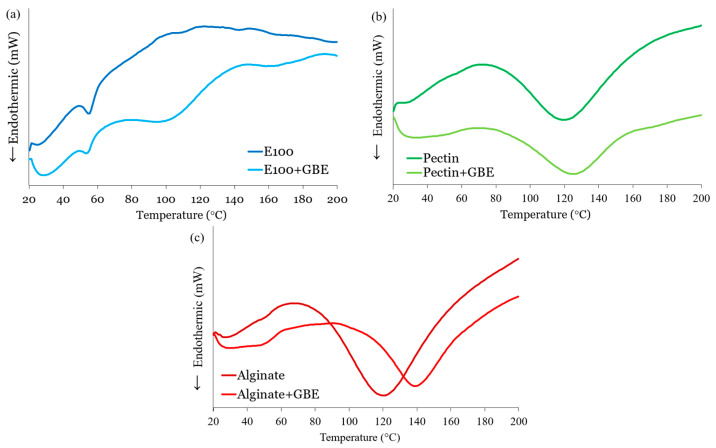
Comparative DSC thermograms of (**a**) E100 loaded and unloaded microparticles, (**b**) Pectin loaded and unloaded microparticles and (**c**) Alginate loaded and unloaded microparticles, loaded and unloaded microparticles.

In order to investigate the thermal properties and stability of the produced microparticles, DSC analyses were performed with empty and loaded microparticles of Eudragit, Alginate and Pectin (Figure 2).

The glass transition temperature (Tg) is a key thermal parameter reflecting the transition of a polymer from a rigid, glassy state to a more flexible, rubbery state. Reported Tg values for sodium alginate generally range from 80 to 120 °C, while pectin exhibits Tg values between 100 and 150 °C, depending on the degree of esterification and moisture content. Eudragit E100 has a Tg around 48 °C, and RS30D around 55 °C. These values provide a reference for interpreting thermal transitions observed in DSC experiments, although slight deviations are expected due to formulation factors and the presence of additives such as GBE.

So, Eudragit polymers generally exhibit similar thermal behavior, with glass transition temperatures ranging from 40 to 65 °C and thermal stability sufficient for pharmaceutical processing techniques such as coating and hot-melt extrusion. According to polymer data tables, Eudragit RS30D has a Tg of approximately 55 °C, and E100 a Tg of approximately 48 °C. RS30D has a slightly higher Tg, implying its polymer matrix remains “glassy” until a higher temperature than E 100. A lower Tg (E 100) means greater flexibility and easier processing at lower temperatures, which is advantageous for immediate-release or taste-masking formulations. Regarding E100 and E100 + GBE microparticles, both formulations show endothermic events related to moisture loss (30–90 °C range), but the E100 + GBE sample exhibits an earlier and more pronounced transition. This suggests increased water availability or weaker water binding, likely due to hydrophilic interactions between GBE constituents and the polymer [25]. In the main thermal transition region (90–160 °C), pure E100 displays broad, complex endothermic behavior, characteristic of structural transitions such as glass transition or early degradation. When GBE is incorporated, these events shift to lower temperatures and become less intense, indicating a plasticizing effect and reduced thermal stability [26]. The presence of GBE likely disrupts E100’s internal structure, enhancing molecular mobility and reducing crystallinity [21].

As for loaded and unloaded pectin microparticles, both display endothermic peaks in the moisture loss region (30–100 °C), with the loaded microparticles showing a broader and more intense peak, indicative of greater water retention through hydrogen bonding between pectin and hydrophilic GBE constituents like polysaccharides and flavonoids (Figure 2b). The main thermal transition in Pectin + GBE occurs at approximately 125 °C, which is slightly higher than the ~120 °C observed for unloaded pectin. Pectin’s high degree of esterification may facilitate hydrophobic interactions with GBE flavonoids, partially offsetting the hydrogen bonding network and lowering the thermal stability. However, unlike E100, in the higher temperature region (~100–160 °C), the thermal transition or decomposition peak shifts to a lower temperature in Pectin + GBE compared to pectin alone. This suggests that GBE acts as a plasticizer here as well, reducing thermal stability by disrupting pectin’s structural order or crystallinity [27].

The presence of endothermic peaks in both loaded and unloaded alginate microparticles indicates typical thermal events such as moisture evaporation and polymer transitions (Figure 2c). The main thermal transition in Alginate + GBE occurs at approximately 140 °C, which is higher than the ~120 °C observed for unloaded alginate. Notably, the Alginate + GBE sample demonstrates broader and higher-temperature endothermic transitions in the 30–100 °C range, suggesting stronger water binding—likely due to interactions between alginate and GBE bioactive compounds, like polyphenols and sugars [26]. Furthermore, the Alginate + GBE microparticles exhibit a shift in the decomposition onset to higher temperatures (~120–160 °C), indicating enhanced thermal stability. This shift implies a potential interaction between GBE and the alginate matrix, such as hydrogen bonding or entrapment, which strengthens the structure [26].

Overall, the incorporation of GBE distinctly affects the thermal properties of each polymer. While Eudragit E100 and pectin exhibit reduced thermal stability due to GBE’s plasticizing and matrix-disrupting effects, alginate shows improved thermal resistance when combined with GBE. These differences reflect varied polymer–extract interactions and compatibility, with implications for the design and stability of nutraceutical delivery systems based on these biopolymers. All these thermal characteristics are critical for selecting appropriate processing conditions. For instance, the lower thermal stability of E100 + GBE may require reduced inlet temperatures during spray drying or modified storage conditions to maintain product integrity.

### 3.2. In Vitro Studies

**Figure 3:**
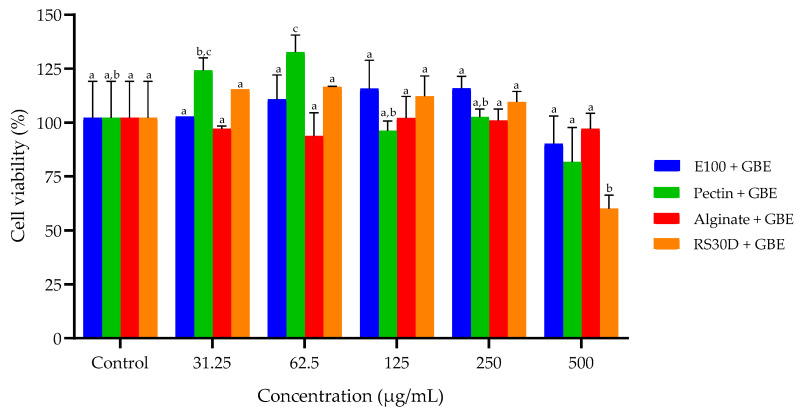
Effect of the spray-dried loaded microparticles on the viability of HT29-MTX cell line after 24 h exposure measured by an MTT assay (*n* = 3), at different concentrations (31.25–500 µg/mL). Different letters (^a,b,c^) indicate significant differences between different concentrations of the same sample (*p* < 0.05).

HT29-MTX cell line was selected as complementary in vitro models of the human intestinal epithelium, given that the microparticles developed in the present study are designed for oral nutraceutical delivery and therefore must be capable of traversing the gastrointestinal environment. Figure 3 summarizes the viability of HT29-MTX cell line after exposure to all the produced microparticles assessed by an MTT assay.

In a previous study, the cytotoxicity of the GBE was evaluated in HT29-MTX, with the authors reporting maintained cell viabilities between 80% and 100% at concentrations ranging from 0.1 to 500 µg/mL [5]. However, at 1000 µg/mL, a marked dose-dependent cytotoxic effect was observed, where viability dropped sharply to 17.02%, highlighting the importance of limiting direct exposure to high doses of bioactive compounds, particularly in sensitive gastrointestinal cell models [5]. Therefore, in the present study, concentrations ranging from 500 to 31.25 µg/mL of each GBE-loaded microparticle produced were evaluated. In general, the results demonstrate that exposure to the developed microparticles did not result in significant reductions in cell viability across most formulations, indicating a favorable biocompatibility profile.

Among the four tested carriers, pectin-based microparticles exhibited the highest cell viability values at multiple concentrations, with statistically significant increases observed at 31.25 and 62.5 µg/mL compared to the control (*p* < 0.05). This outcome suggests that pectin may confer enhanced protection or stabilization of the encapsulated bioactive compounds [24,28]. Additionally, pectin’s inherent mucoadhesive and biocompatible properties may support improved interaction with epithelial cells, favoring cellular metabolic activity [28]. These findings are consistent with previous reports showing that pectin-based nanoparticles reduced the cytotoxicity of resveratrol in HT29-MTX cell lines when compared to the free compound, with cell viabilities maintained above 81% [29]. Such results reinforce the notion that pectin not only serves as a safe encapsulating matrix but also contributes to modulating the release profile of bioactive compounds, thereby reducing their potential cytotoxic effects on intestinal epithelial models [29]. Interestingly, the concentration of 62.5 µg/mL resulted in significantly higher viability compared to 125 and 500 µg/mL, suggesting a biphasic dose–response relationship. The same happened in San Hipólito-Luengo et al. [30] study were resveratrol promoted HT-29 cell growth at lower concentrations (1–10 μmol/L) and inhibited HT-29 cell growth when in higher concentration (50–100 μmol/L), which the authors attributed to the induction of cell death through apoptosis and necrosis. Regarding the RS30D + GBE microparticles, while generally well tolerated, a notable drop in cell viability at 500 µg/mL was shown, suggesting potential cytotoxicity at elevated concentrations. This could be due to the physicochemical properties of the RS30D polymer, which may influence the interaction between the encapsulated extract and the cellular membrane, or result in slower degradation and accumulation effects [31]. Conversely, both E100 and alginate-based microparticles maintained relatively stable and acceptable viability across all concentrations tested, confirming their suitability as inert and protective carriers for oral delivery.

Comparing the present study with Teixeira et al. [5] study, the current data reinforces the importance and effectiveness of microencapsulation in improving the safety profile of phytochemical-rich extracts. All formulations in this study were produced using mild, physical encapsulation techniques, preserving the chemical integrity of the goji extract while enabling controlled release. Notably, none of the tested microparticle formulations caused a decrease in viability comparable to that seen with the free extract at high doses, even at the maximum concentration tested (500 µg/mL). This suggests that encapsulation reduces cytotoxic risks by modulating release kinetics and minimizing direct contact with cellular membranes, underscoring the importance of both encapsulating material and dosage in modulating cellular responses and ensuring formulation safety.

## 4. Conclusions

The present study provides compelling evidence that the produced GBE-loaded microparticles, especially using pectin as encapsulating agent, can significantly improve its safety profile while preserving its biological functionality. The tested microparticles maintained cell viability across physiologically relevant concentrations, and showed adequate morphology and strong bioactivity, as shown by antioxidant and antiradical evaluations, suggesting that the encapsulated formulations are well-tolerated by intestinal epithelium and can serve as effective carriers for targeted delivery of bioactive compounds. Given the well-documented antioxidant potential of goji berries, these GBE-loaded microparticles offer strong potential for use in functional foods and dietary supplements—whether as powder ingredients for smoothies, yogurts, and shakes, or as standalone nutraceuticals. From a nutritional standpoint, although there is no established recommended daily intake (RDI) for total antioxidants, it is generally accepted that diets rich in natural antioxidants (e.g., >1000 mg/day of polyphenol-rich foods) contribute to the prevention of oxidative stress-related chronic diseases. The GBE-loaded microparticles developed in this study could serve as a valuable delivery platform to help individuals meet these intake goals, particularly in populations with poor antioxidant-rich dietary patterns. Therefore, these findings support the potential application of GBE-loaded microparticles as a functional ingredient in health-oriented food products or as a dietary supplement, offering a safe, stable, and effective means to deliver natural antioxidants. Future studies, including in vivo evaluations and product development trials, are warranted to further validate their efficacy and potential health benefits in real-life applications.

## Figures and Tables

**Table 1:** Production yield and average size of all the produced microparticles Results are expressed as mean ± standard deviation (*n* = 3).

	Production Yield (%)	Average Size (µm)
**E100**	2.8 ± 0.1	3.3 ± 2.1
**E100 + GBE**	26.6 ± 8.0	2.9 ± 0.7
**Alginate**	13.9 ± 0.7	3.2 ± 1.0
**Alginate + GBE**	40.3 ± 7.0	3.6 ± 1.6
**Pectin**	21.7 ± 1.1	3.5 ± 2.7
**Pectin + GBE**	38.1 ± 3.0	4.1 ± 1.0
**RS30D**	1.3 ± 0.1	4.4 ± 2.0
**RS30D + GBE**	20.1 ± 6.5	1.9 ± 0.9

**Table 2:** Total phenolic content (TPC), ferric reducing antioxidant power (FRAP) and antiradical activity (DPPH radicals scavenging capacity) of the GBE and the produced microparticles Results are expressed as mean ± standard deviation (*n* = 3).

	TPC	FRAP	DPPH
	mg GAE/g	µmol FSE/g	mg TE/g
**GBE**	23.87 ± 1.16 ^e^	105.97 ± 8.20 ^f^	10.25 ± 0.81 ^d^
**E100**	3.53 ± 0.58 ^a^	5.10 ± 0.99 ^a^	0.94 ± 0.27 ^a^
**E100 + GBE**	9.31 ± 1.18 ^c^	25.96 ± 7.04 ^b,c^	4.01 ± 1.38 ^c^
**Pectin**	3.60 ± 0.25 ^a^	18.50 ± 1.86 ^b^	1.05 ± 0.24 ^a^
**Pectin + GBE**	8.14 ± 0.50 ^b^	43.20 ± 5.26 ^d^	4.14 ± 1.74 ^c^
**Alginate**	2.61 ± 0.47 ^a^	20.87 ± 3.51 ^b,c^	1.06 ± 0.49 ^a^
**Alginate + GBE**	7.85 ± 0.46 ^b^	42.16 ± 6.16 ^d^	3.33 ± 0.80 ^b,c^
**RS30D**	9.61 ± 0.81 ^c^	26.59 ± 3.30 ^c^	1.77 ± 0.49 ^a,b^
**RS30D + GBE**	15.51 ± 1.16 ^d^	59.83 ± 7.96 ^e^	3.50 ± 1.78 ^c^

GAE, Gallic acid equivalents; FSE, Ferrous sulphate equivalents; TE: Trolox equivalents. Different letters (^a,b,c,d,e,f^) in the same column indicate significant differences between mean values (*p* < 0.05).

## Data Availability

The original contributions presented in the study are included in the article; further inquiries can be directed to the corresponding author.
